# Safety, Tolerability, and Pregnancy Outcomes Following Gravibinan Administration in Women at Risk of Miscarriage: A Multi-institutional Retrospective Study

**DOI:** 10.7759/cureus.88063

**Published:** 2025-07-16

**Authors:** Khadija Bano, Seema Muzammil, Atfa Muzamil, Khair-un-Nisa Nizam, Naila Ehsan, Shahida Magsi, Maria Rasheed, Atif A Hashmi

**Affiliations:** 1 Obstetrics and Gynecology, Al-Tibri Medical College, Karachi, PAK; 2 Obstetrics and Gynecology, Zambia Hospital, Lahore, PAK; 3 Obstetrics and Gynecology, Al Haseeb Maternity Clinic, Lahore, PAK; 4 Obstetrics and Gynecology, Indus Medical College, Tando Muhammad Khan, PAK; 5 Internal Medicine, Sindh Government Urban Health Center, Karachi, PAK; 6 Obstetrics and Gynecology, Saleem Medical Center, Quetta, PAK; 7 Obstetrics and Gynecology, Chandka Medical College, Larkana, PAK; 8 Obstetrics and Gynecology, District Headquarters (DHQ) Hospital, Muzaffargarh, PAK; 9 Pathology, Liaquat National Hospital and Medical College, Karachi, PAK

**Keywords:** gravibinan, high-risk pregnancies, miscarriage, pain, redness

## Abstract

Objective

Gravibinan is commonly used to prevent or manage miscarriage in high-risk pregnancies. However, data on its safety profile in real-world clinical settings remains limited. Hence, this study aimed to evaluate the safety and tolerability of Gravibinan in pregnant women at risk of miscarriage (two or more consecutive pregnancy losses) before the 20th week of gestation.

Methodology

This retrospective observational study was conducted in multiple tertiary care settings to assess the safety profile of Gravibinan in pregnant women. The study duration was about five months from January 2025 to May 2025. The inclusion criteria for the study were pregnant women between the ages of 18 and 45 years who had received a minimum of one dose of Gravibinan during their current pregnancy. The demographic details, such as age, height, weight, body mass index (BMI), and gestational age at enrollment, were documented. Cases with a history of miscarriage were included in the study. Gravibinan was administered intramuscularly at a dose of either 1 ml or 2 ml, initiated between the 5th and 7th week of gestation in patients with a history of recurrent miscarriage. Treatment was continued once or twice weekly until the end of the first trimester. Additionally, immediate local reactions along with any systemic side effects, were also recorded.

Results

Out of 534 pregnant women, the mean age of participants was 30.72 ± 5.90 years, with a mean gestational age of 13.55 ± 11.40 weeks at the time of enrollment. The majority, 408 (76.4%), received a 2 ml dose weekly. Local side effects like pain at the injection site at 214 (40.1%) and redness at 86 (16.1%) were mostly mild. Systemic side effects included nausea at 130 (24.3%), dizziness at 58 (10.9%), and vomiting at 70 (13.1%), with very few cases of serious symptoms like respiratory distress or vaginal bleeding at 8 (1.5%) each. Fetal outcomes were favorable, with a mean birth weight of 4.60 ± 1.74 pounds and an average Apgar score of 7.07 ± 1.03. Most women, 530 (99.3%), had normal postpartum health and satisfactory reproductive health in follow-up.

Conclusion

Gravibinan seems to be safe and well-tolerated in pregnant women at risk of miscarriage, with minimal adverse effects and positive maternal and fetal outcomes. Statistically significant associations were found between dosage and side effects such as injection site pain, swelling, dizziness, and nausea.

## Introduction

Miscarriage, described as the spontaneous loss of pregnancy before 20 weeks of gestation, affects around 10-20% of clinically recognized pregnancies and represents a significant clinical and emotional burden for affected individuals and healthcare systems alike [1]. Multiple etiological factors contribute to miscarriage, with chromosomal abnormalities responsible for approximately 50-60% of first-trimester losses (before 13 weeks), anatomical defects around 10-15%, infections and endocrine disorders each accounting for roughly 10-15%, and immune dysregulation in about 5-10%, with the distribution varying between early and late miscarriages [2]. Therapeutic strategies to prevent miscarriage in high-risk populations (two or more consecutive pregnancy losses) have long been a focus of obstetric research.

The two primary sex hormones that support and maintain pregnancy in its initial phases are progesterone and estrogen [3]. In addition to altering the secreted substances within the endometrium linings and facilitating effective implantation of embryos, the hormones such as progesterone and estrogen also improve vascularization [4]. They also keep the gestational sac intact and assist the growing fetus. One of the most common characteristics reported in research is that any decrease in the level of progesterone can contribute to recurrent miscarriage. Around 10-12 weeks of gestation, the placenta takes over hormone production, ensuring continued support for fetal development [5].

This artificial variant of progesterone, called progestin, helps to treat evolved uterine cancer, hormonal imbalances that cause unusual bleeding, and aids early pregnancy continuation while also preventing aberrant uterine lining hypertrophy [6].

The yearly miscarriage rate in Pakistan is approximately 10 to 12%, based on a total of 26.9 births per 1000 population, which is comparable to the global miscarriage rate estimated at 10-15% of clinically recognized pregnancies [7,8]. Limited knowledge, lack of education, inadequate nutrition for mothers, malnourishment, low socioeconomic position, prior stillbirths, mother’s age, inadequate healthcare systems, hormonal imbalance, and other associated issues all contribute to the nation's considerable incidence of miscarriages [9]. There is insufficient information to determine Pakistan's precise miscarriage rate. In India, the incidence rate of spontaneous recurrent miscarriages is 32%, causing parental sorrow and negatively impacting maternal health [10].

Gravibinan, a synthetic substance having progestogenic and immune-modulatory qualities, has drawn interest as a possible miscarriage prophylaxis tool, especially in women who have experienced repeated miscarriages. Other supportive methods like progesterone suppositories are commonly used, providing context for evaluating Gravibinan’s potential role in early pregnancy support [11]. Preliminary studies suggest that Gravibinan may support early pregnancy maintenance by enhancing endometrial receptivity and modulating immune responses at the maternal-fetal interface [12]. However, concerns remain regarding its safety profile, especially in the delicate context of early gestation, where both maternal and fetal health are highly susceptible to pharmacological interventions.

Pro-inflammatory cytokines linked with miscarriages and the amount of blocking factor (PIBF), which is triggered by progesterone, have an impact on the immune responses [13]. To prevent miscarriages, the current trial used Gravibinan, the most dependable supply of progesterone. Gravibinan indications include preventing miscarriages in high-risk pregnancies [14].

Given the limited data on Gravibinan’s safety, a comprehensive evaluation is necessary to inform clinical practice. This article reviews the available evidence regarding the safety of Gravibinan in patients with a higher-than-normal likelihood of experiencing a miscarriage during pregnancy, aiming to clarify its risk-benefit profile and guide its potential use in obstetric care.

## Materials and methods

This retrospective observational study was conducted across multiple healthcare centers, including Indus Medical College & Hospital, Tando Mohammad Khan; Al-Tibri Medical College and Hospital, Malir, Karachi, Sindh; District Headquarters (DHQ) Hospital in Muzaffargarh; Chandka Medical College & Hospital, Larkana; Saleem Medical Complex, Quetta; Ghulam Haider Family Clinic, Gujranwala; and Al Haseeb Clinic & Maternity Home, Lahore, to evaluate the safety profile of Gravibinan in pregnant women. The study duration was about five months, from January 2025 to May 2025. The ethical approval was obtained from the relevant ethics committee (Research Ethical Committee, Sindh Government Urban Health Center, Karachi; approval number UHC/KAR/48-50-25) prior to the initiation of the study, guaranteeing that all research operations adhered to accepted ethical norms and regulations. The recruitment process was conducted over a defined period of five months across more than one healthcare facility, such as antenatal clinics, maternity wards, or obstetrics outpatient departments. A total of 534 pregnant women between the ages of 18 and 45 who were attending routine antenatal check-ups and who had received at least one dosage of Gravibinan, considered at risk for miscarriage typically included those with a history of one or more previous miscarriages, advanced maternal age (e.g., over 35), known hormonal imbalances (such as low progesterone), underlying medical conditions (e.g., diabetes, thyroid disorders), anatomical abnormalities of the uterus, or lifestyle and environmental risk factors (such as smoking or high stress) were included in this study. To be eligible for the study, females must also have access to their full clinical records, which must include follow-up information and recorded fetal outcomes. Exclusion criteria comprised pregnant women with missing or incomplete medical records, a history of significant comorbidities unrelated to pregnancy (e.g., chronic renal disease, malignancy), or pregnancies affected by known fetal congenital abnormalities, such as neural tube defects (e.g., spina bifida), congenital heart defects, cleft lip or palate, limb deformities, or chromosomal abnormalities like Down syndrome.

The data was collected using a proforma (see Appendices). The data collection method included extracting significant clinical, demographic, and obstetric data from patient records. Demographic details like age, height, weight, body mass index (BMI), gestational age at enrollment, and past history of abortions were documented.

Gravibinan is produced and marketed by OBS Pharma Pvt. Ltd. in Pakistan. It is typically available as a 250 mg/5 mg oil-based intramuscular ampule, stored under controlled conditions, and administered per clinician guidance.

Gravibinan was administered intramuscularly at a dose of either 1 ml or 2 ml, initiated between the 5th and 7th week of gestation in patients with a history of recurrent miscarriage. Treatment was continued once or twice weekly until the end of the first trimester. Gravibinan was administered to pregnant women with a history of recurrent miscarriage as a preventive measure to support pregnancy maintenance. The standard treatment protocol involved a 1 ml dose administered once weekly. However, the dosage and frequency were adjusted based on clinical assessment. In such cases, treatment was intensified to 2 ml per dose or administered twice weekly to provide enhanced hormonal support during the critical early stages of gestation. The therapy was continued until the completion of the first trimester (12 to 14 weeks), the period during which the risk of miscarriage is highest. Moreover, immediate local reactions, for instance, pain, redness, swelling, lump formation, and warmth at the injection site were documented, along with any systemic side effects, such as dizziness, palpitation, vomiting, nausea, cough, respiratory distress, and bleeding per vaginum. The mode of delivery was recorded for all participants and categorized as either vaginal or cesarean to assess pregnancy outcomes in relation to Gravibinan administration. Fetal outcomes were evaluated through birth weight, Apgar scores, and mode of delivery, while maternal outcomes involved postpartum health status and reproductive health in subsequent pregnancies.

This study assessed both independent and dependent variables. The independent variable was the administration of Gravibinan, with its dosage and frequency. The dependent variables included the incidence of adverse effects, encompassing both maternal and fetal outcomes. They also involved assessing local reactions at the injection site, systemic side effects, and delivery outcomes, offering a thorough overview of the potential impacts of Gravibinan use during pregnancy.

Data analysis

The data was entered and analyzed using IBM SPSS Statistics for Windows, version 23 (IBM Corp., Armonk, NY, USA). The descriptive statistics, such as demographic and clinical variables, were reported as means and standard deviations, and frequencies and percentages were used for categorical data. A chi-square test was used to find out the association of local and systemic side effects and maternal and fetal outcomes with Gravibinan dosage administered. A p value < 0.05 was considered statistically significant.

## Results

Out of a total of 534 female participants selected for the study, the mean age of the participants was 30.72 ± 5.90 years, with an average body weight of 65.93 ± 9.18 kg and a mean height of 5.30 ± 0.20 feet. The calculated mean BMI was 27.01 ± 3.46 kg/m². At the time of enrollment, the mean gestational age was 13.55 ± 11.40 weeks. Among the participants who had experienced previous pregnancy losses, the average number of previous miscarriages was 1.78 ± 0.89. On average, the participants received 9.57 ± 5.30 injections during the treatment course. In terms of fetal outcomes, the mean birth weight was recorded as 4.60 ± 1.74 pounds, while the average Apgar score at birth was 7.07 ± 1.03. (Table 1).

**Table 1:** Demographic details of pregnant women (n=534)

Variable		Mean ± SD
Age (Year)		30.72 ± 5.90
Weight (Kg)		65.93 ± 9.18
Height (Ft)		5.30 ± 0.20
BMI (kg/m2)		27.01 ± 3.46
Gestational age at enrollment (weeks)		13.55 ± 11.40
If yes, number of previous miscarriages		1.78 ± 0.89
Total injections administered		9.57 ± 5.30
Fetal outcomes	Birth weight (Ibs)	4.60 ± 1.74
	Apgar score	7.07 ± 1.03

The results from Table 2 showed that out of the total participants, 266 women (49.8%) were primiparous, while 268 (50.2%) were multiparous. Regarding the status of the current pregnancy, 434 (81.3%) experienced a threatened miscarriage, and 100 (18.7%) were receiving prophylaxis for recurrent pregnancy loss. Among the participants, 88 (16.5%) were diabetic, whereas 446 (83.5%) were non-diabetic. Hypertension was present in 38 women (7.1%), and absent in 496 (92.9%). In terms of ethnicity, 122 participants (22.8%) were Urdu-speaking, 145 (27.1%) were Sindhi, 128 (23.9%) were Punjabi, and 139 (26.1%) belonged to the Pathan or Balochi ethnic groups. Regarding the dosage administered, 126(23.6%) women received 1 ml of the injection, while 408 (76.4%) received 2 ml. Most participants, 472 (88.4%), received the injections weekly, whereas 62 (11.6%) received them twice weekly. For mode of delivery, 360 women (67.4%) had a vaginal delivery, and 174 (32.6%) underwent cesarean section.

**Table 2:** Demographic, clinical, and treatment characteristics of study participants

Variable		n	%
Gravidity parity	Primiparity	266	49.8
	Multiparity	268	50.2
History of termination of pregnancy or miscarriage	Threatened miscarriage (loss of pregnancy typically before 20 weeks of gestation)	434	81.3
	Prophylaxis for recurrent pregnancy loss	100	18.7
Diabetes	Yes	88	16.5
	No	446	83.5
Hypertension	Yes	38	7.1
	No	496	92.9
Ethnicity	Urdu-speaking	122	22.8
	Sindhi	145	27.1
	Punjabi	128	23.9
	Pathan/Balochi	139	26.1
Dosage administered	1 ml	126	23.6
	2 ml	408	76.4
Injection frequency	Weekly	472	88.4
	Twice weekly	62	11.6
Mode of delivery	Vaginal	360	67.4
	Cesarean	174	32.6

The results presented in Table 3 show that a variety of local and systemic adverse effects were reported following Gravibinan administration. Pain at the injection site was noted by 214 (40.1%) women, while 320 (59.9%) did not experience any pain. In terms of pain intensity, the majority reported mild pain 408 (76.4%), followed by moderate 108 (20.2%), and severe pain 18 (3.4%). Redness or erythema was observed in 86 (16.1%) cases, whereas 448 (83.9%) showed no such reaction. Swelling at the injection site was reported by 84 (15.7%) participants, while 450 (84.3%) did not experience swelling. Lump formation was a rare occurrence, reported by only 4 (0.7%) women, with 530 (99.3%) showing no signs of lumps. Warmth at the injection site was also uncommon, occurring in 10 (1.9%) participants and absent in 524 (98.1%). Regarding systemic side effects, dizziness post-injection was reported by 58 (10.9%) women, and 476 (89.1%) did not experience it. Palpitations occurred in 16 (3.0%) participants, whereas 518 (97.0%) did not report this symptom. Cough was experienced by 28 (5.2%) women, and 506 (94.8%) did not experience any coughing. Both fits and respiratory distress were noted in 8 cases each (1.5%), with 526 (98.5%) not exhibiting these symptoms. Gastrointestinal side effects included nausea in 130 (24.3%) participants and vomiting in 70 (13.1%), while the remaining 404 (75.7%) and 464 (86.9%), respectively, did not report these symptoms. Bleeding per vaginum was also observed in 8 (1.5%) cases, whereas 526 (98.5%) did not experience this. Menstrual irregularities were noted by 17 (3.1%) participants, while 517 (96.9%) had no complaints. Fetal abnormalities were reported in 14 (2.6%) cases, while 520 (97.4%) had no such findings.

**Table 3:** Local and systemic adverse effects reported following Gravibinan administration

Variable		n	%
Pain	Yes	214	40.1
	No	320	59.9
Intensity	Mild	408	76.4
	Moderate	108	20.2
	Severe	18	3.4
Redness erythema	Yes	86	16.1
	No	448	83.9
Swelling	Yes	84	15.7
	No	450	84.3
Lump formation	Yes	4	0.7
	No	530	99.3
Warmth at the injection site	Yes	10	1.9
	No	524	98.1
Side effects post-injection dizziness	Yes	58	10.9
	No	476	89.1
Palpitation	Yes	16	3.0
	No	518	97.0
Cough	Yes	28	5.2
	No	506	94.8
Fits	Yes	8	1.5
	No	526	98.5
Respiratory distress	Yes	8	1.5
	No	526	98.5
Nausea	Yes	130	24.3
	No	404	75.7
Vomiting	Yes	70	13.1
	No	464	86.9
Bleeding per vaginum	Yes	8	1.5
	No	526	98.5
Menstrual irregularities	Yes	17	3.1
	No	517	96.9
Fetal abnormalities	Yes	14	2.6
	No	520	97.4

Pain was significantly more prevalent in the 2 ml group 259 (63.5%), compared to the 1 ml group 41 (32.8%), with a highly significant association (χ² = 37.656, p < 0.001). Similarly, swelling was reported in 76 (18.6%) of the 2 ml group versus only 8 (6.3%) in the 1 ml group, showing a significant difference (χ² = 10.948, p = 0.001). Dizziness following injection was notably higher in the 1 ml group 38 (30.2%), compared to 20 (4.9%) in the 2 ml group, which was statistically significant (χ² = 63.430, p < 0.001). Nausea was also significantly more common in the 1 ml group 58 (46.0%), than in the 2 ml group 72 (17.6%) (χ² = 42.113, p < 0.001). No significant association was observed between dosage and redness/erythema (χ² = 1.416, p = 0.234), vomiting (χ² = 0.024, p = 0.876), bleeding per vaginum (χ² = 2.508, p = 0.113), menstrual irregularities (χ² = 0.329, p = 0.566), or fetal abnormalities (χ² = 0.761, p = 0.684), indicating these outcomes were not significantly influenced by the dose of Gravibinan administered, as reported in Table 4.

**Table 4:** Association of local and systemic adverse effects with Gravibinan dosage administered

Variables		Gravibinan dosage administered			
		1 ml (n=126)	2 ml (n=408)	Pearson Chi-square	p value
Pain	Yes	41 (32.8%)	259 (63.5%)	37.656	<0.001
	No	85 (67.2%)	149 (36.5%)		
Redness erythema	Yes	16 (12.7%)	70 (17.2%)	1.416	0.234
	No	110 (87.3%)	338 (82.8%)		
Swelling	Yes	8 (6.3%)	76 (18.6%)	10.948	0.001
	No	118 (93.7%)	332 (81.4%)		
Side effects post-injection dizziness	Yes	38 (30.2%)	20 (4.9%)	63.430	<0.001
	No	88 (69.8%)	388 (95.1%)		
Nausea	Yes	58 (46.0%)	72 (17.6%)	42.113	<0.001
	No	68 (54.0%)	336 (82.4%)		
Vomiting	Yes	16 (12.7%)	54 (13.2%)	0.024	0.876
	No	110 (87.3%)	354 (86.8%)		
Bleeding per vaginum	Yes	0 (0.0%)	8 (2.0%)	2.508	0.113
	No	126 (100.0%)	400 (98.0%)		
Menstrual irregularities	Yes	5 (4.0%)	12 (2.9%)	0.329	0.566
	No	121 (96.0%)	396 (97.1%)		
Fetal abnormalities	Yes	4 (3.2%)	10 (2.4%)	0.761	0.684
	No	122 (96.8%)	398 (97.5%)		

The findings from Table 5 reveal that the vast majority of participants, 530 women (99.3%), had a normal postpartum maternal health status, while only 4 women (0.7%) experienced complications. Regarding reproductive health in subsequent pregnancies, the majority of participants, 516 (96.6%), experienced a satisfactory or normal course, indicative of healthy pregnancies without significant complications. A smaller proportion, 10 (1.9%), reported no issues or complications, categorized as "Nil," while 8 (1.5%) reported minor issues that were considered "Not Significant" and did not adversely affect the pregnancy outcomes.

**Table 5:** Postpartum maternal health and reproductive outcomes in subsequent pregnancy

Variable		n	%
Postpartum maternal health status	Normal	530	99.3
	Complications (such as infections, hemorrhage, or other postpartum disorders)	4	0.7
Reproductive health in subsequent pregnancy	Satisfactory/normal	516	96.6
	Nil (no issues or complications reported)	10	1.9
	Not significant (no significant issues)	8	1.5

## Discussion

This study aimed to assess the safety of Gravibinan use in pregnant women, with a particular emphasis on maternal side effects, fetal outcomes, and overall drug tolerability. The results demonstrated that Gravibinan was generally well-tolerated, with most side effects being mild and resolving on their own.

In the present study, pain at the injection site emerged as the most frequently reported local reaction, noted in 40.1% of participants, though predominantly of mild severity. Incidences of redness and swelling were relatively low, observed in about 16% of cases, while serious local reactions such as lump formation (0.7%) and warmth (1.9%) were rare, indicating good local tolerability. These findings align with previous research on intramuscular hormonal treatments during pregnancy, where injection site discomfort is reported in approximately 30-50% of cases, typically not necessitating medical intervention [15].

Several progesterone-based medications are commonly used to support the early stages of pregnancy, including Gravibinan, Utrogestan, and Cyclogest [16]. One of the meta-analyses reported no significant increase in congenital abnormalities or adverse drug reactions with vaginal progesterone [17]. The present study was consistent with the above-reported studies and found few severe adverse effects with Gravibinan. Common issues included pain (40.1%), nausea (24.3%), and vomiting (13.1%), while serious complications like fits, respiratory distress, and lump formation were rare (<2%). In contrast, the present study on Gravibinan (an injectable combination of estradiol valerate and hydroxyprogesterone caproate) reported favorable outcomes, with 67.4% vaginal deliveries and normal postpartum health in 99.3% of women. Also, 96.6% had a satisfactory reproductive course in subsequent pregnancies.

The present study on Gravibinan, demonstrated favorable outcomes in high-risk pregnancies, with a total miscarriage rate of approximately 19.6%, aligning closely with or outperforming outcomes from prior hormonal supplementation studies. In comparison, a retrospective cohort evaluating oral estradiol and dehydroepiandrosterone (DHEA) supplementation in women with low early-pregnancy estradiol levels reported miscarriage rates of 21.2% and 17.5%, respectively, with DHEA showing statistically significant efficacy (p = 0.038) [18].

In our retrospective cohort of 534 Gravibinan-treated women (mean age 30.7 ± 5.9 years; BMI 27.0 ± 3.5 kg/m²; average 9.6 injections) at risk of miscarriage, outcomes were favorable-mean birth weight of 4.6 lb, Apgar score of 7.1, with 67% vaginal delivery; local site reactions and systemic side effects were mostly mild (pain 40%, nausea 24%, vomiting 13%, fetal abnormalities 2.6%). These results align closely with the randomized clinical trial by Devine et al., which compared intramuscular progesterone (every third day) versus vaginal progesterone alone in frozen embryo transfer cycles [19]. In that study, the IM group achieved significantly higher live birth rates (54% vs 37%) and lower miscarriage rates (~19% vs 27%) compared to vaginal administration [19].

Regarding side effects, local injection site reactions were observed in the present study, including pain 214 (40.1%), redness 86 (16.1%), and swelling 84 (15.7%), alongside systemic symptoms such as nausea 130 (24.3%) and vomiting 70 (13.1%). These results are consistent with previous studies, which identified nausea and abdominal bloating as common adverse effects associated with progesterone use, irrespective of the formulation (oral micronized progesterone or dydrogesterone) [20]. However, dizziness was reported in 10.9% of participants in our study, which was lower compared to the higher incidence of dizziness (giddiness) reported by Shaikh et al., particularly in the micronized progesterone group (22.86%) [20]. Moreover, drowsiness was significantly more frequent in the micronized progesterone group (61.43%) compared to the dydrogesterone group (31.43%) [20].

Additionally, the current findings demonstrated that almost all participants, 530 (99.3%), maintained normal postpartum maternal health, and most reported normal reproductive health during subsequent follow-ups. These outcomes are consistent with prior research supporting the role of progesterone in enhancing pregnancy continuation and live birth rates [21-25], confirming that progesterone supplementation effectively reduces miscarriage rates and improves both maternal and fetal outcomes without significant long-term complications [20].

This study has a few limitations. This study was retrospective and relied on existing clinical records, which may be subject to documentation bias or incomplete data. Additionally, the lack of a control group limits the ability to infer causality. Prospective, controlled studies are recommended to further validate the safety and efficacy of Gravibinan. Routine monitoring for side effects should continue, and standardized protocols for dosing and follow-up should be established to optimize maternal-fetal outcomes.

## Conclusions

This study concluded that Gravibinan has a favorable safety profile in pregnant women at risk of miscarriage. The majority of participants tolerated the drug well, with only mild to moderate local injection site reactions such as pain and redness, and relatively low rates of systemic side effects, including nausea and dizziness. Statistically significant associations were found between dosage and side effects such as injection site pain, swelling, dizziness, and nausea. However, no significant differences were observed in serious outcomes such as fetal abnormalities between the two dosage groups. Overall, Gravibinan use was generally well-tolerated, with minimal adverse effects on maternal and fetal health.

## Appendices

Patient questionnaire form

1. Patient Demographic & Anthropometric Information

Age (in years): ______

Weight (in Kg): ______

Height (in Ft): ______

BMI (kg/m²): ______

Gestational Age at Enrollment (in weeks): ______

2. Obstetric History

Parity:

______Primiparity

______Multiparity

Number of Previous Miscarriages (if any): ______

History of Termination of Pregnancy or Miscarriage:

_____Yes    ______No

Threatened Miscarriage (before 20 weeks):

______Yes    ________No

Prophylaxis for Recurrent Pregnancy Loss Taken:

_______Yes    ________No

3. Medical History

Diabetes:

_______Yes    ______No

Hypertension:

______Yes    ______No

4. Ethnicity

______Urdu Speaking

_______Sindhi

________Punjabi

________Pathan / Balochi

5. Injection Therapy Details

Total Injections Administered: ______

Dosage Administered:

______1 ml    ______2 ml

Injection Frequency:

_______Weekly    _______Twice Weekly

6. Fetal Outcomes

Birth Weight (in lbs): ______

APGAR Score: ______

Fetal Abnormalities:

______Yes    _______No

7. Mode of Delivery

________Vaginal

________Cesarean

8. Local Injection Site Reactions

Pain at Injection Site:

______Yes    ________No

If Yes, Intensity of Pain:

_____Mild    _______Moderate    _______Severe

Redness/Erythema:

______Yes    _______No

Swelling:

_____Yes    _______No

Lump Formation:

______Yes    _____No

Warmth at Injection Site:

_______Yes    _______No

9. Systemic Side Effects Post Injection

Dizziness:

____Yes    _____No

Palpitation:

______Yes    _____No

Cough:

_____Yes    ______No

Fits:

_____Yes    _____No

Respiratory Distress:

_____Yes    _______No

Nausea:

______Yes    _____No

Vomiting:

_____Yes    _____No

Bleeding Per Vaginum:

_____Yes    _____No

Menstrual Irregularities:

_____Yes    _____No

10. Postpartum and Reproductive Health

Postpartum Maternal Health Status:

______Normal

______Complications (e.g., infections, hemorrhage, etc.)

Reproductive Health in Subsequent Pregnancy:

_______Satisfactory/Normal

________Nil (No issues or complications reported)

_______Not Significant (No significant issues)
